# Patient-Centered Care for Adolescents and Young Adults with an Uncertain or Poor Cancer Prognosis: A Secondary Analysis of What Is Needed According to Patients, Caregivers, and Healthcare Providers

**DOI:** 10.3390/curroncol32020058

**Published:** 2025-01-21

**Authors:** Milou J. P. Reuvers, Vivian W. G. Burgers, Eveliene Manten-Horst, Kim Messelink, Elsbeth J. H. M. van der Laan, Winette T. A. van der Graaf, Olga Husson

**Affiliations:** 1 Department of Medical Oncology, Netherlands Cancer Institute—Antoni van Leeuwenhoek, 1000 SE Amsterdam, The Netherlands; m.reuvers@nki.nl (M.J.P.R.); w.vd.graaf@nki.nl (W.T.A.v.d.G.); 2 Department of Medical Oncology, Erasmus MC Cancer Institute, Erasmus University Medical Center, 3015 GD Rotterdam, The Netherlands; 3 Dutch AYA Care Network, 3511 DT Utrecht, The Netherlands; 4 Medical Oncology, Radboud University Medical Center, 6525 GA Nijmegen, The Netherlands; 5 Department of Surgical Oncology, Erasmus MC Cancer Institute, Erasmus University Medical Center, 3015 GD Rotterdam, The Netherlands; 6 Department of Public Health, Erasmus MC Cancer Institute, Erasmus University Medical Center, 3015 GD Rotterdam, The Netherlands

**Keywords:** patient-centered care, adolescents and young adults, oncology, uncertain or poor prognosis

## Abstract

Patient-centered care focuses on aligning healthcare with a person’s values and preferences to support their health and life goals. This approach is especially crucial among adolescents and young adults (AYAs—with a primary cancer diagnosis between the ages of 18 and 39) facing an uncertain or poor cancer prognosis (UPCP), whose care needs differ from those undergoing curative treatment. This study aims to gain insights from AYAs with a UPCP, their informal caregivers, and healthcare professionals (HCPs) to define optimal patient-centered care and identify barriers to its implementation. We conducted semi-structured interviews with 46 AYAs, 39 informal caregivers, and 49 HCPs from various clinical backgrounds. Findings highlighted the need of AYAs for an equal relationship with HCPs and active involvement in decision-making, alongside tailored information addressing their unique challenges. Informal caregivers expressed the need for information to support patients while preferring a minimal focus on themselves. HCPs noted the necessity for specialized training to meet the specific needs of AYAs with a UPCP, reporting difficulties in providing tailored support due to the disease’s uncertainties. This study’s results can lead to improved healthcare for this population and enhance educational modules for HCPs, equipping them to better support AYAs facing a UPCP.

## 1. Introduction

**Figure 1:**
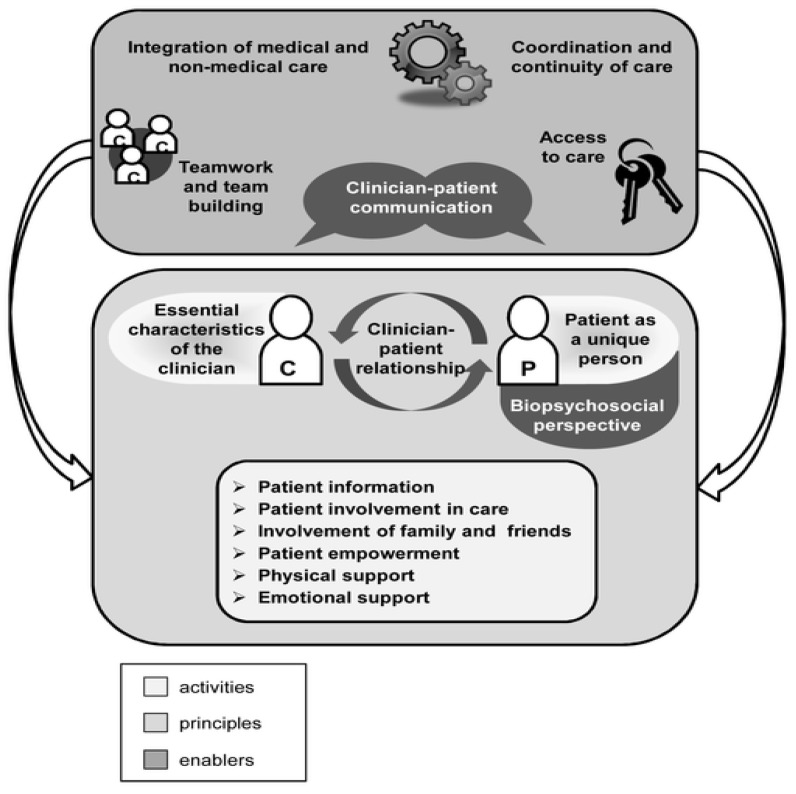
The integrative model of patient-centeredness by Scholl et al. [2].

Over the years, the healthcare system has evolved, with an emphasis on the individual and what is meaningful to them. Patient-centered care (PCC) implies that “an individuals’ values and preferences are elicited and once expressed, it guides all aspects of their healthcare and supports their realistic health and life goals” [1]. A literature review by Scholl and colleagues identified 15 domains as components of PCC (Figure 1). These domains encompass principles (i.e., fundamental propositions, which lay the foundation of PCC), enablers (i.e., elements, which foster PCC), and activities (i.e., specific PCC behavior) essential for providing tailored care to patients. They address the characteristics of healthcare professionals (HCPs), holistic patient perspectives, effective communication between patients and physicians, adequate support, patient and informal caregiver involvement, and the necessary conditions of the healthcare system [2]. While PCC has the potential to enhance the quality of healthcare, implementing it can be challenging. As it focuses on a specific individual, tailoring is required to fit a specific situation. This puts pressure on an already overburdened system, where time is limited and the workload is high. Currently, there is not an optimal comprehensive care pathway for addressing all psychosocial needs. It can be difficult to determine who is responsible for taking care of certain issues, and long-term care is not always well coordinated or available [1,3].

A good example of PCC is the care provided to adolescents and young adults (AYAs—those diagnosed with cancer for the first time between 18 and 39 years old in the Netherlands). These patients report age-related issues, including difficulties in establishing an identity, impaired self-esteem, social isolation, issues with fertility, and financial hardship. Age-specific care programs have been developed to meet patients’ unique needs in order to provide appropriate care tailored to the developmental phase of an AYA patient, including a focus on psychosocial aspects [4,5]. Dutch AYA care is nurse-led and utilizes specific educational modules for HCPs [6]. Many AYA cancer patients report similar priorities during treatment: being able to live a normal life, accomplishing developmental milestones, and spending time with those they care about [7]. For the majority of AYAs, age-specific care is provided during curative treatment or survivorship. However, 15 to 20 percent of AYA patients live with an uncertain or poor cancer prognosis (UPCP)—that is, those with advanced cancer, without a reasonable hope of cure, who will likely die prematurely from their disease but do not face an immediate threat of premature death. Because this group is a small subset within AYA oncology, little is known about what these patients perceive as important in their healthcare [8].

Current programs do not specifically focus on the needs of this recently defined heterogeneous subgroup of AYA cancer patients. Our recent studies show that the uncertainty experienced by these patients results in distinct disease trajectories and coping mechanisms when compared to other patients, potentially leading to unique care needs [8,9]. Moreover, HCPs also encounter difficulties when caring for this group and their informal caregivers report various challenges in their daily life due to their role as caregiver [10]. Although optimal PCC requires a holistic approach, no research has combined the perspectives of the AYAs with a UPCP, their informal caregivers, and HCPs. Since the literature on AYAs with a UPCP is scarce, a qualitative approach can be used for the in-depth exploration of the unique experiences and needs associated with PCC of patients, informal caregivers, and HCPs to further enhance the patient-centered care model for this specific AYA group.

This paper provides a secondary analysis of the input from the earlier studies conducted on AYA patients with UPCP, informal caregivers, and their HCPs [9,10,11], and uses this to expand Scholl’s model of PCC for this specific patient group.

The aim was to synthesize insights from patients, informal caregivers, and their HCPs to provide an overview of the preferences and needs related to delivering and receiving care for this unique patient group and those around them. These data can be used to provide practical guidelines for the current healthcare, highlighting specific considerations for AYA patients alongside general factors essential for effective care. The focus is on what preferences and needs reported by the three stakeholders are specific to AYA patients, but this paper also considers generic factors that may still be important in providing appropriate and sufficient care.

## 2. Materials and Methods

### 2.1. Sample and Procedure

In this study, data from the INVAYA-study were used, in which interviews were conducted with AYAs with a UPCP, their informal caregivers, and HCPs. The INVAYA-study aimed to gain insight and understand experiences of the three subgroups through in-depth interviews. The methodology used to conduct the interviews with AYA patients, their informal caregivers, and HCPs was reported in previous articles [9,10,11]. Patients were signed up via their healthcare provider and were invited by the researcher (VB—psychologist) to participate. Patients were allowed to invite their informal caregivers, who were contacted by the researcher after 1–2 weeks. HCPs were invited via purposive sampling. Interviews were planned and conducted either face to face or via Microsoft Teams due to the COVID-19 pandemic. The interview guides for all three stakeholder groups are reported in the Appendix A (Table A1, Table A2 and Table A3). In total, 46 AYA patients with a UPCP participated in the interviews. Subsequently, 39 informal caregivers were interviewed, including 13 partners, 12 parents, 7 friends, and 7 siblings. Lastly, 49 HCPs with different specializations were interviewed. The interviews with patients and informal caregivers primarily focused on their healthcare needs and the impact of disease. The HCPs were mainly addressing the challenges they face while caring for AYA patients with a UPCP. This study was approved by the Institutional Review Board of the Antoni van Leeuwenhoek hospital in Amsterdam, the Netherlands (IRBd20-205).

### 2.2. Data Analysis

For the primary analysis of the interview transcripts, elements of the grounded theory of Corbin and Strauss were used [12]. For primary and secondary analysis, we used reflexive thematic analysis by Braun and Clarke was used [13]. In the primary analysis, data from the interview transcripts concerning the impact of the disease, healthcare experiences, and challenges in caring for these patients were coded by two reviewers (MR, VB) [9,10,11]. Additionally, to achieve the aim of this article, a secondary analysis of these codes was conducted to identify those that were related to healthcare. These were then categorized as either specific to (or to have more impact at the age of) AYA patients or as more generic themes applicable to all (cancer) patients (MR, WG). This was discussed until consensus was reached. Afterwards, codes were categorized into themes, and all themes were divided among the fifteen domains of Scholl’s model. Themes could be added to more than one domain if they were related to multiple aspects of PCC. This method aligns with the Framework Analysis by Ritchie and Spencer [14]. QSR NVIVO was used to conduct qualitative analysis [15]. Descriptive analyses were conducted using SPSS v29.0.

## 3. Results

The sociodemographic characteristics of AYA patients with a UPCP, their informal caregivers, and HCPs are reported in Table 1. Codes that were age-specific among AYA patients with UPCP, informal caregivers, and their HCP are presented in Table 2. The associated quotes are shown in Table A4. General codes are reported in Table A5.

Some topics in the tables are negatively phrased. These topics have not yet been sufficiently implemented in the current system of care to ensure good PCC. Once they are properly applied, they function as part of the section they are a part of (principle, enabler, or activity).

### 3.1. AYA-Specific Care Needs

PCC among AYAs with a UPCP should focus on addressing all impacted domains of life without feeling resistance (e.g., using soft or hard drugs, sexuality or fertility), as these topics are often overlooked or undiscussed by HCPs due to the poor prognosis. Furthermore, they want tailored information and support, which is an important factor of PCC. Both these factors should be applicable to their situation: premature mortality and a young age. AYA patients with a UPCP report a need for clear communication from their HCPs regarding their disease, prognosis and end-of-life care. This can help them to make age-related life decisions.

PCC for informal caregivers should focus on receiving appropriate information regarding the specific issues and situation of the AYA patient. This information can help informal caregivers to understand the AYAs, give them insight into how to support the AYAs adequately, and enable them to provide emotional support. Furthermore, this can help them to be able to support the patients with making decisions. Regarding care for themselves, partners of the AYAs want to be able to discuss fertility with an HCP but also receive information on how to cope with the disease and treatment as an informal caregiver.

HCPs are motivated to provide the best care for these patients but face multiple challenges when trying to apply PCC. They perceive AYAs with a UPCP as a challenging group and would like specific training on how to provide the best possible care. Furthermore, they express a need for emotional support, as they empathize with this group and find it burdensome to take care of young patients who might die prematurely. HCPs struggle with balancing between asking enough questions and providing enough support, without belittling the AYA. The tailored information (e.g., life expectancy and possibilities to start a new study, job, or hobby) requested by patients and informal caregivers is not always available, making it impossible for HCPs to provide this information and advice. Sensitive or personal topics can be difficult to discuss or do not seem appropriate due to poor prognosis, leading to some of them not being brought up by HCPs. In addition, providing PCC can be challenging when managing the dynamics of different stakeholders in the room.

### 3.2. General Care Needs

Aside from the age-specific preferences, several general themes emerged from the interviews (Table A5). The impact of the general topics could be more extensive for AYA patients with a UPCP; however, these needs could also be existent among other cancer patients, such as AYAs treated with curative intent and older adult patients. Nevertheless, it is important to address these topics, as these do seem important to AYA patients with a UPCP.

HCPs who invest time and are familiar with the AYAs’ situation, are empathetic, can be trusted, and adopt a holistic approach are important for PCC in AYAs patients with a UPCP. To receive appropriate care, patients adjust their behavior accordingly to ensure HCPs will maintain their efforts. Patients want the opportunity to discuss alternative treatment options, as well as the end of life and their prognosis, at a time that is appropriate to them. An essential aspect of PCC is knowing who to approach in case of questions or the need for additional support. Furthermore, they would like information on which support is available for them. Shared decision-making is another important aspect. Lastly, it is important that HCPs ask specific questions that are relevant and tailored to the AYAs’ situation, compared to asking general questions.

Informal caregivers expect knowledge and empathy from HCPs. PCC should focus on the quality of life and the possibilities of the patients. Informal caregivers do not want to draw too much attention to themselves. They want to know what support is available and who to turn to in case of any questions. It is important to have regular check-ins by HCPs regarding their own well-being and to ensure they feel acknowledged and supported. Additionally, they want information on how to support the patients. When necessary, they would like their own psychological trajectory independent of the patient. They want the end of life to be discussed, but only when unavoidable.

When HCPs provide PCC, they mention that being unable to give patients any certainty is challenging. Balancing being open versus maintaining a professional distance is difficult, and they also struggle with knowing what needs to be addressed. They feel like they lack the knowledge that is expected (e.g., estimating whether a patient’s reaction is normal or pathological, or information about alternative therapies). They feel unaware of available support, and it can be difficult to assess what is necessary. HCPs aim to avoid putting too much pressure on the patient and seek opportunities to empower them where possible. They also fear taking away all patients’ hope, and can struggle to work with multiple HCPs, as it can be unclear to determine which responsibility one HCP has.

## 4. Discussion

**Figure 2:**
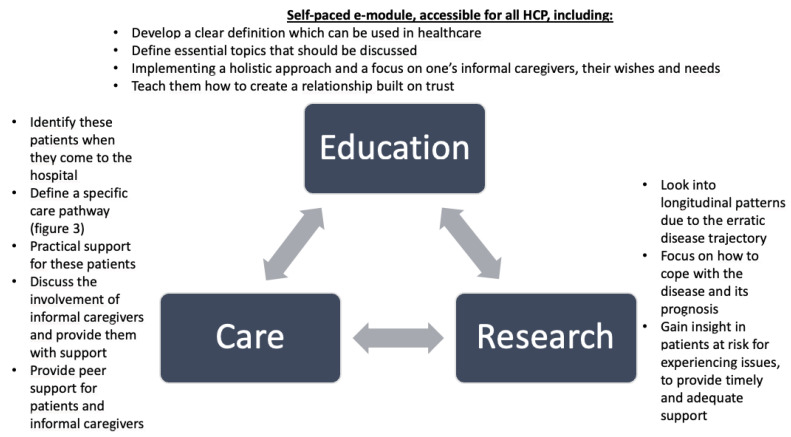
Self-learning healthcare model of care for AYAs with a UPCP.

This study highlights the distinct experiences and challenges faced by AYAs with a UPCP, their informal caregivers, and HCPs. We aim to guide adaptations in the healthcare for AYAs, focusing on three key pillars: education, healthcare, and research (Figure 2). By creating a “self-learning healthcare system”—where these pillars continuously give each other input to generate development—a solid foundation for these three stakeholders can be established, ensuring their voices are heard and their needs are addressed. To meet the needs of AYAs with a UPCP and their informal caregivers in clinical practice, three steps are essential: (1) to ensure HCPs are aware of these patients’ and informal caregivers’ potential needs, are confident in conducting need assessments, provide tailored care, and have knowledge regarding available services and referral pathways; (2) to integrate AYAs and their informal caregivers into the current care model and allocate appropriate time for them; (3) to empower AYAs to actively express their healthcare needs and preferences to receive sufficient care [16].

The first key pillar is education, aimed at equipping HCPs to identify AYA patients with a UPCP, understand key discussion topics, and know how to provide the best care for these patients and their informal caregivers. This can be achieved by developing an e-learning, focusing on patients’ unique challenges: being young and dealing with premature death, loss, uncertainty (e.g., which milestones can one accomplish), and feeling alone within the healthcare setting and among peers. HCPs should learn to initiate discussions of topics like fertility, sexuality, and premature death, and consider how to discuss life decisions and wishes with patients. Building trust, sharing decision-making, and learning how make appropriate referrals are essential for PCC in AYAs with a UPCP and their informal caregivers. These factors are important among all cancer patients; however, among AYAs with a UPCP, these are crucial. Having a relationship built on trust can guide patients through the uncertainty and the healthcare system, in which most of them are unfamiliar [17,18]. With proper training, HCPs can become more confident in their own abilities, causing less of a burden and making this type of healthcare more manageable [19,20].

**Figure 3:**
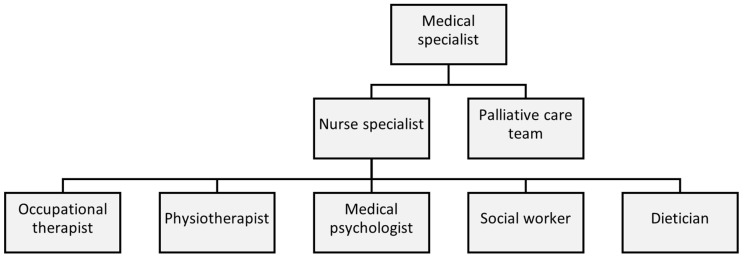
Care pathways when caring for AYAs with UPCP.

To effectively integrate care for AYAs with a UPCP, as mentioned in pillar two, we can learn from the Dutch AYA care model and its care pathway, designed by the Dutch AYA Care Network [21]. This provides HCPs with a checklist for essential questions and actions. However, it requires adaptations for AYA patients with a UPCP, starting with a tailored checklist used to define this group. Then, these patients should be referred to multiple disciplines for additional support (Figure 3). It can be challenging for an healthcare provider and AYA to talk about the end of life, which can complicate the referral to a palliative care team [22,23]. However, this is necessary to obtain a holistic understanding of one’s needs and quality of life aspects, which should then guide the medical trajectory, rather than solely focusing on the remaining time left. Preferably, all AYAs with a UPCP should be referred at some time to a psychologist and/or palliative care physician specialized in AYA patients. A similar AYA-specific palliative care model, developed by the Princess Margaret Cancer Centre, has shown promising results, including improvements in symptom management and end-of-life planning [24]. To implement an effective referral network, it is crucial to clearly define the responsibilities of each healthcare provider. Since AYA patients may not always be aware of the available support, HCPs should inform them about and refer them to the appropriate resources, including emotional and psychological support such as Acceptance and Commitment Therapy (ACT) or Managing Cancer and Living Meaningfully (CALM) therapy [25,26,27,28]. Additionally, practical support should be addressing needs such as employment, finance, diet, exercise, childcare, sexual health and fertility, complementary care, epilepsy management, and support for informal caregivers [29,30]. Since most AYAs with a UPCP experience an erratic disease trajectory, additional support may not always be necessary, indicating the need for prompt referral when issues arise. According to pillar three, the support for these patients should not only focus on attempting to resolve all difficulties: this patient group is struggling with issues that may not be resolvable, or that are appropriate in this abnormal situation. Empowering patients or reinforcing their positive characteristics may also be an appropriate method to adequately support them. A peer support network can help to normalize emotions, offer advice, and provide mutual support. Furthermore, support for informal caregivers is limited and often requires a psychiatric diagnosis in the Netherlands, while more guidance and accessible support are needed. We have to learn from other diseases and initiatives to explore the possibilities within the Dutch healthcare.

Clinical practice can generate topics for further research in this field. Currently, we are conducting research to longitudinally and exploratively examine what challenges AYAs with a UPCP are dealing with and what needs may exist, as they face a constantly changing disease trajectory. We aim to gain information on how these patients cope with their disease and their prognosis by including factors such as hope, meaning and purpose, and resilience. By gaining insight into the patients that are more prone to problems and establishing at which point these issues occur in this trajectory, appropriate support can be provided in a timely manner. This knowledge can complement the e-module, allowing the care for this group to be adjusted accordingly. This can also result in the healthcare system being less burdened: by gaining more insight into the most common issues and identifying the groups most affected by them, we can provide more targeted support. This ensures that actions are taken only where necessary, reducing the workload.

Our research contributes to shaping the AYA care in the Netherlands for patients with a UPCP, incorporating different perspectives to illuminate both preferences and barriers. However, this study has several limitations. Conducting secondary analysis can lead to hindsight bias, but may also lead to the absence of relevant data because of adherence to codes analyzed in the previous studies. The interview scripts for all three stakeholders were different and specifically tailored, and were also collaboratively developed with input from stakeholders, as detailed in previous articles. For example, interviews with informal caregivers focused on the challenges of supporting someone with a UPCP. As a result, the code “essential characteristics” was not identified in their transcripts, leaving that section incomplete. This deductive coding process, which derived codes directly from the data underscores the need for further research into the preferences and needs of the stakeholders. Furthermore, the input of informal caregivers of AYAs with a UPCP to PCC was limited in this article since the interviews were focusing more on their caregiver burden, and it is possible that their perspectives were not thoroughly integrated into the model. This highlights the need for research on the PCC needs of this unique group of informal caregivers. The sample used is large for qualitative research, and so all views expressed can be regarded well. A limitation of this study is that it is difficult to generalize the findings to non-Dutch healthcare settings. It is possible that patients living in a different type of society (individualistic versus collective) have different needs. It is advised to perform this study in other countries to examine the discrepancies. The literature shows that cultural differences exist regarding disclosure of diagnosis and prognosis, the implementation of traditional healthcare, decision-making [31,32,33], the discussing end of life, and the involvement of informal caregivers [32]. Cultural differences emphasize the need for an open attitude that approaches patients as individuals and takes a personalized approach, as highlighted by this study. Culture is an important aspect for the AYA-population and the role of loved ones within the disease trajectory is culturally dependent. Additionally, AYA patients consist of both generation X, Y (millennials) and Z, who might have different needs regarding communication. It is important to examine these different needs and to use these when implementing patient-centered care [34].

## 5. Conclusions

To provide PCC for AYA patients with a UPCP, it is essential to make adaptations to the current AYA care pathway and focus specifically on AYAs with a UPCP, as this study has shown that these patients and their informal caregivers have unique and age-specific care needs, and HCPs report challenges in providing this specific care. When one’s life expectancy is uncertain, we argue for a focus on quality of life which revolves around the activities or goals that are important to someone in order to provide direction in the uncertainty rather than planning life solely based on the remaining time. The results of this study highlight that it is important to provide informal caregivers with more access to care by giving them a contact person to whom they can request additional support or ask questions to. Furthermore, integrating PCC for AYAs with a UPCP into the current healthcare system seems to require educating HCPs about the information and communication needs of the AYAs and empowering the AYAs to express their needs and navigate the healthcare setting more effectively. Identifying AYA patients with a UPCP is the initial step to aligning with their healthcare needs, with a focus on holistic healthcare and issues arising in essential life domains. Finally, it is important to establish an effective referral network with defined responsibilities of each healthcare provider to optimize access, coordination, and continuity of care.

## Figures and Tables

**Table 1:** Sociodemographic characteristics of the participants.

	Patients (n = 46) N (%)	Informal Caregivers (n = 39) N (%)	HCP (n = 49) N (%)
Mean age (±SD) at interview Mean age at diagnosis	33.4 (6.3) 29.6 (4.8)	41.8 (13.77) n/a	46.6 (9.5) n/a
Sex Female Male	29 (63.0) 17 (37.0)	26 (66.7) 13 (33.3)	42 (85.7) 7 (14.3)
Marital status Married/with partner Single	38 (82.6) 8 (17.4)	38 (97.4) 1 (2.6)	n/a n/a
Living situation * With partner With children Alone Other	16 (34.8) 19 (41.3) 7 (15.2) 2 (4.3)	35 (89.7) 19 (48.8) 2 (5.1) 1 (2.6)	n/a n/a n/a n/a
Educational level Secondary education or less Secondary vocational education Applied university University	4 (8.7) 16 (34.8) 16 (34.8) 10 (21.7)	1 (2.6) 13 (33.3) 19 (48.7) 6 (15.4)	n/a n/a n/a n/a
Employment status * Full-time Self-employed Part-time Unemployed Sick leave/disabled Retired Student Other	11 (23.9) 2 (4.3) 7 (15.2) 1 (2.2) 23 (50.0) 0 (0) 3 (6.5) 0 (0)	16 (41.0) 0 (0) 15 (38.5) 3 (7.7) 2 (5.1) 4 (10.3) 0 (0) 1 (2.6)	n/a n/a n/a n/a n/a n/a n/a n/a
Relationship to the patient Partner Parent Friend Sibling Clinical nurse specialist Medical oncologist Neurologist Psychologist Social worker Gynecologist Surgeon Clinical occupational doctor Occupational therapist Psychiatrist Pulmonary physician Radiation oncologist Rehabilitation physician Support consultant Urologist	n/a n/a n/a n/a n/a n/a n/a n/a n/a n/a n/a n/a n/a n/a n/a n/a n/a n/a n/a	13 (33.3) 12 (0.8) 7 (17.9) 7 (17.9) n/a n/a n/a n/a n/a n/a n/a n/a n/a n/a n/a n/a n/a n/a n/a	n/a n/a n/a n/a 10 (20.4) 9 (18.4) 7 (14.3) 5 (10.2) 5 (10.2) 3 (6.1) 2 (4.1) 1 (2.0) 1 (2.0) 1 (2.0) 1 (2.0) 1 (2.0) 1 (2.0) 1 (2.0) 1 (2.0) 1 (2.0)
Type of cancer Low-grade glioma Sarcoma Breast cancer Lung cancer Melanoma Cervical cancer Other	15 (34.7) 7 (15.2) 6 (13.0) 6 (13.0) 3 (6.5) 2 (4.3) 6 (13.0)	n/a n/a n/a n/a n/a n/a n/a	n/a n/a n/a n/a n/a n/a n/a

* participants were able to select multiple answers.

**Table 2:** Age-specific codes among AYAs with a UPCP, informal caregivers, and their HCPs according to the model of patient-centeredness by Scholl et al. [2].

	AYA	Informal Caregiver	HCP
**Principles**			
Essential characteristics of the clinician	▪ Feel like the clinician works harder because they are young patients ▪ Would like the HCPs to have AYA-specific knowledge		▪ Have difficulty relating to the patient when there is a large age gap ▪ Have a need for specialized training on how to deal with these patients ▪ Wanting support as HCPs empathize with AYAs, which is challenging
Clinician–patient relationship	▪ Want to feel equal (2.1) ▪ Do not want to be treated pedantically ▪ Want to be approached as an adult ▪ The large age gap makes them view HCPs as parent-like—causes difficulty discussing all topics	▪ The large age gap makes them view HCPs as parent-like—causes difficulty discussing all topics (2.2)	▪ Find it challenging to balance offering help and patronizing the patient ▪ Have difficulty dealing with young, male patients (less open, want to prove themselves, difficult to connect to them, not always honest about the impact)
Patient as a unique person	▪ Feel odd in healthcare setting ▪ Statistics often do not apply to them	-	▪ Cannot give age-specific advice and information, as this is not available
Biopsychosocial perspective	▪ Fertility is not discussed (4.1) ▪ Have a need for information and support for children (4.2) ▪ Would prefer to spend time discussing specific issues: making life decisions while being of young age and facing premature death, and also dealing with loss, uncertainty and confrontation because of the disease ▪ Want to receive support to live a meaningful life	▪ Have a need for information and support for children	▪ Unclear if patients have the desire to start a family ▪ Have difficulty discussing specific topics as they do not want to shock or force patients ▪ Find it more difficult to give diagnosis when patients have young children
**Enablers**			
Clinician–patient communication	▪ Want to make own decisions, despite age and lack of experience with healthcare setting ▪ Do not want parents to be involved, unless specifically discussed (5.1) ▪ The large age gap makes them view HCP as parent-like—causes difficulty discussing all topics		▪ Not wanting to come across as pedantic and treat a patient as adult ▪ Unclear if patients have the desire to start a family ▪ Have difficulty discussing specific topics as you do not want to shock or force patients ▪ Find it difficult to discuss fertility as an ethical discussion might develop ▪ Difficulty discussing end of life as there is no clear information on this topic
Integrating medical and non-medical care	▪ Have difficulty discussing certain topics (such as use of drugs or sexuality)		▪ Find it difficult to discuss fertility as an ethical discussion might develop ▪ Challenging to discuss romantic relationships and offer support
Teamwork and teambuilding			▪ It can be unclear who to refer to ▪ It can be unclear from whom the appropriate information can be obtained
Access to care	▪ Are unaware of support services available ▪ Do not feel at ease at the hospital as they are so young	▪ Perceive a lack of fertility preservations at hospital	
Coordination and continuity of care			
**Activities**			
Patient information	▪ Have a need for more time and information regarding fertility preservations ▪ Perceive a lack of information about diagnosis at such young age	▪ Have a need for information about how others deal with the same situation (10.1) ▪ Want information for their children to be able to engage them	▪ Have a need for a list of available support and information, to be able to communicate this with these patients.
Patient involvement in care	▪ Want to make their own decisions, despite age and lack of experience with the healthcare setting		
Involvement of family and friends	▪ Not being completely honest when parents are present during consultations (12.1) ▪ Do not want parents to be involved, unless specifically discussed		▪ Have difficulty with patients who are not honest to protect their caregivers, which makes it difficult to know how one is doing ▪ Challenging when patients do not involve parents at all ▪ Find it difficult to manage patients and parents who have different opinions. ▪ Feel frustration in the consultation room when parents are overprotective
Patient empowerment	▪ Young patients are not familiar with the healthcare system and are unsure of how to navigate through it		▪ Difficulty with AYAs who can be overly prepared, ask many questions, have a need for control and treatment preference ▪ Difficulty with AYAs who do to not ask for help as much and rather do things themselves, resulting in support not being provided in a timely or optimal manner ▪ Would like AYAs to participate in support groups ▪ Want to obtain an AYA book for AYAs
Physical support			
Emotional support	▪ Want to receive support from someone who has been in the same situation ▪ Do not relate to elderly patients ▪ Do not relate to curative peers	▪ Want to receive support from someone who has been in the same situation (15.1)	▪ Find it burdensome to work with young patients who will die prematurely and want support ▪ Want support as HCPs empathize with AYAs, which is challenging

## Data Availability

Data are not available due to privacy restrictions.

## Appendix A

**Table A1:** Interview guide used among AYA patients with UPCP.

Questions	Probes
Could you please tell me about your experience with the hospital as a young patient with cancer?	Could you please tell me what goes well? Could you please tell me what kind of problems you are running into?
Do you or did you have unmet needs regarding information or support?	If so, could you please elaborate? (It was not assessable; you didn’t find the right support or didn’t know where to find it) Could you please give me an example of a situation in which you did not even notice you needed information or support until you received the information or support? Who is your contact person within the hospital for specific questions? Could you please elaborate?
How would you describe the relationship with your primary healthcare professional? (Possible to repeat these questions multiple times for different healthcare professionals)	Could you please give me an example that showcases this well? What aspects are you satisfied about? What would you like to see differently? What questions should your healthcare professional ask you or what should they notice about you to know how you are really doing?
With whom of the healthcare professionals do you feel free to discuss whatever you want?	Could you please elaborate? (What topics do you discuss, differences between healthcare professionals) Are there any taboo topics you do not want to discuss with your healthcare professional(s)? Could you please elaborate?
What do you need from your healthcare professional when talking about end of life?	What are your needs regarding communication about end of life? (When and how) Do they communicate clearly about which phase of the disease you are in currently?

**Table A2:** Interview guide used among informal caregivers.

Questions	Probes
How did the disease of your AYA impact your life? (e.g., daily life, major life decisions)	Education, employment, finances, hobbies, being independent, social life, family life
Do you think that the impact would have been different if your AYA would get better/would be curatively treated?	Could you please elaborate? Could you please give me an example that showcases this well? What are you afraid of?
In what way did the disease of your AYA impact your relationship? Did your relationship change and, if so, how? Who among you has changed?	Sexuality, intimacy, roles/relationships, equality, tasks, financial independency, communication
How do you cope with the impact of the disease on your life? What would help you to deal with this more effectively? How could your AYA best support you in this regard?	
How do you experience the support you receive? How do you think you and your AYA can best support/help each other?	To what extent, in what way, frequency, and from whom do you experience support? What could they do better? Is there support you are missing/ have been missing? What advice do you have for your support system? What would be the best way to support you right now?
How has your perspective on the future changed as a result of the diagnosis? How do you perceive the future? What are your needs regarding communication about the future?	How do you see the future? What would you prefer based on the current situation? What concerns you the most? What are you afraid of? Did this change over time? With whom can you (not) communicate about this? How do you talk about it then? With whom would you like to communicate about it?

**Table A3:** Interview guide used among HCP for AYA patients with UPCP.

Questions	Probes
Could you please tell what difficulties you ran into when delivering care to AYAs with a UPCP?	Could you please take me through a specific case which describes these experiences in the best way? How do you currently cope with this challenge?
Do you experience any difficulties when discussing some topics with AYAs with a UPCP?	Could you please elaborate? Could you please give me an example that showcases this well? What are you afraid of?
What do you perceive as challenging patient groups within this AYA population?	Can you give an example that visualizes the challenges you come across when dealing with this patient group(s)? How do you currently cope with this patient groups?

**Table A4:** Quotes derived from the interviews.

Related Domain	Quote Number	Quote
Clinician–patient relationship	2.1	Recently, I have experienced our relationship to be unequal. Thus, her being superior to me and [my partner]. That it was like a parental meeting where parents reprimanded us about what we should and should not do. And I believe it is very important to be on the same level (*patient, F, 28, sarcoma*)
	2.2	For the first time, we had a doctor our age and the other one retired. And the relationship, right away it is sort of different. The conversations are sort of different. And that is okay […]. But often it makes discussions easier or something (*partner, F, 34*)
Biopsychological perspective	4.1	I receive treatment which can cause infertility. And we have never talked about that. And I know that that happened because they think I will not be cured. So why think about having children? But that could have been discussed. Both by me and them. Maybe in this case, the responsibility is with the clinician. (*patient, F, 30, sarcoma)*
	4.2	I have missed support and information about dealing with young children. I was in the pulmonary department, where fairly few women, let alone young women were treated Also, there was no support for practical issues arising with having little children: what do you do when you have to go to the hospital, or when you do not feel well? (*patient, F, 41, lung cancer*)
Clinician–patient communication	5.1	So, I got a call on Friday afternoon at 5:00 PM but I could not answer. So, in the meantime, my neurologist called my father. And I did not like that, because I thought: this is about me, I am 23 years old, and my dad does not decide about that. (*patient, F, 23, brain tumor*)
Patient information	10.1	No, you really do not know what to expect. From: okay, when the side-effects are intense, what can I expect? You know, you just get very vague answers […]. So, for that matter I would like to hear from others: okay, what can we expect, you now? What did you have? Yeah, I find it difficult that, that that is not possible. And you can find it often via a Facebook-page. Even though I often think you also read horror-stories on there and sometimes you do not want to. (*partner, F, 33*)
Involvement of friends and family	12.1	I am not sure if elaborating on it works for me, because I do not want to be 100% honest about how shit I am feeling when my parents are there […]. But also, no critical questions are asked. (*patient, F, 23, brain tumor*)
Emotional support	15.1	But then you get to, […], the [hospital] and you see a lot of people there. And, well, it is not something that is soothing, it is only that you know that you are not in this by yourself. And, yeah, that is like […], that a lot of people are dealing with some sort of suffering. And in that regard, it is comforting, in that way. You are not happy with it, but it is comforting in that way. (*father, M, 55*)

**Table A5:** General care needs among AYAs, informal caregivers and their HCP according to the model of patient-centeredness by Scholl et al. [2].

	AYA	Informal Caregiver	HCP
**Principles**			
Essential characteristics of the clinician	▪ HCP have enough time and specific knowledge about the diagnosis ▪ HCP are prepared and has read the patient’s file ▪ HCP have empathy, humor and open attitude	▪ HCP have enough time and specific knowledge about their diagnosis ▪ Is prepared and has read a patient’s file ▪ Want to get the feeling HCP are trying their best ▪ Prefer HCP knowing you, being taken seriously and understood ▪ Receiving honest information	▪ Struggle what questions to ask to make sure they are not perceived as curious ▪ Challenging that they are not able to give any certainty to the patient
Clinician–patient relationship	▪ Do not feel equal to the HCP ▪ Trying to be a good patient: little complaining, do as told, do not ask questions, talk about second opinions or discuss all topics ▪ A regular check-in would be helpful ▪ Experience a relationship in which they can trust the HCP	▪ Regular check-in by nurse specialist would be preferred and makes them feel acknowledged ▪ Are unsure if they should blindly follow the HCP	▪ Have difficulty balancing proximity versus being at professional distance ▪ Mention patients that are difficult to work with (angry, critical, never satisfied, psychiatric patients, who keep asking ‘why me’) ▪ Find it difficult to discuss topic early on, as no relationship has been developed
Patient as a unique person	▪ There is not always a focus on what the patient prioritizes/is important to them	▪ Prefer a focus on what matters to the patient ▪ Prefer a focus on what a patient still can do, not only limitations ▪ Prefer a focus on patient as an individual and do not mention not-applicable statistics	▪ Do not know what patients expect from treatment ▪ Do not know what information one wants about prognosis ▪ Have difficulty to determine what topics a patient should raise and what to initiate ▪ Challenging to deal with religious patients who cannot discuss all topics
Biopsychosocial perspective	▪ Perceive a lack of holistic view and only focus on the clinician’s own specialism ▪ There is not always a focus on comorbidities and side-effects ▪ Want end-of-life discussions in home setting, to have it sink in	▪ Perceive a lack of holistic view and only focus on the clinician’s own specialism ▪ Prefer in some cases that the HCP would ask them how they are doing ▪ Have a need for organizations who can help with financial decisions and arrangements	▪ Have difficulty to determine if a patient’s response is normal or problematic, and a need for psychological training to be able to put this into perspective
**Enablers**			
Clinician–patient communication	▪ Want honest and clear communication: understandable language and having to rephrase ▪ Want to be able to e-mail HCP ▪ Want everything to be discussed and not having to look things up themselves ▪ Ask specific, not too general questions, and not discuss topics a patient is not engaged in ▪ HCP should mention what support is available ▪ HCP often perceive scan more positive than patients do ▪ Tend to share less information if HCP is not empathetic ▪ As patients deal with an uncertain disease, they are unsure what to discuss with HCP	▪ Prefer to receive information from the HCP and not having to look things up online ▪ Costs a lot of energy receiving incorrect information or having poor communication with the hospital ▪ Challenging to discuss death-related topics ▪ Want to discuss death-related topics ‘when the time is right’	▪ Having little time to properly discuss all domains of a patient’s life ▪ Have a fear of asking too few questions ▪ Do not want to force patients to discuss difficult topics (e.g., dying) ▪ Are unsure if they are asking enough and appropriate questions ▪ Have a fear of giving patients false hope ▪ Wonder if they have been clear enough when a patient is (too) positive ▪ Have a need for support on how to give bad news
Integrating medical and non-medical care	▪ Perceive a lack of implementation of alternative medicine ▪ Need a clinician to listen to patients when there is a need for alternative medicine use ▪ Perceive a lack of support for going back to work, financial decisions and what you can apply for as a cancer patient ▪ Alternative and complementary treatments are not brought up	▪ Perceive a lack of implementation of alternative medicine ▪ Need a clinician to listen to patients when there is a need for alternative medicine use ▪ There is mainly attention on the medical part of the disease ▪ Perceive a lack of information on how to cope with the disease	▪ Mention not having any knowledge about alternative medicine ▪ Find it difficult when a patient does not disclose alternative medicine use ▪ Feel uncertain whether a patient wants to discuss sexuality with them
Teamwork and teambuilding	-	▪ Want a team that is specialized in this diagnosis and have different HCP work on your case ▪ Hospital should try and fix their mistakes, once they have happened	▪ Difficult to work together with a team, as there is little between HCP ▪ Responsibilities can be unclear when multiple HCP are involved ▪ Have difficulty to hand over care to the nurse specialist
Access to care	▪ Find it difficult who to turn to in case of questions, as the physician does not have time to answer all of them ▪ Challenging to find out what support is available and where to find it ▪ Want a nurse specialist who is available for questions ▪ Want to be able to e-mail HCP ▪ Prefer a nurse specialist to be the link between patient and physician and be available for questions	▪ Find it difficult who to turn to in case of questions, as physician does not have time to answer all of them ▪ Challenging to find out what support is available and where to find it ▪ Prefer to be closer to the hospital and be treated at smaller hospitals, as they are more intimate ▪ Struggle with a hospital that is far away, as this takes a lot of time ▪ Appointments are often delayed or canceled	▪ Have difficulty to find appropriate care for patients with an erratic disease trajectory ▪ Are unsure of what support is available for specific issues (e.g., sexuality) ▪ Referral can be difficult since not all support is reimbursed ▪ It can be challenging to receive information about the patient from other HCP, which makes applying appropriate care difficult
Coordination and continuity of care	▪ Want to stick to one physician and only switch once a patient request this ▪ Do not want to share their story with multiple HCP ▪ Would like one case manager who is familiar with their situation	▪ Struggle with a hospital which is far away, as this takes a lot of time ▪ Receiving results and time between appointments can be long, meaning they do not know when to ask questions ▪ Appointments are often delayed or canceled	▪ Perceive they often do not have all treatment details, making them feel unprepared ▪ Have difficulty to determine treatment plan as the prognosis will not change ▪ Find it burdensome if patients want to be treated elsewhere or are treatment-shopping
**Activities**			
Patient information	▪ Want to talk about specific topics once they are inevitable ▪ Are not sure if they would like information about the prognosis ▪ HCP should ask if they should talk about the prognosis ▪ Wanting to know what is going to happen ▪ They experience a rush through consultations, as HCP seem to think a patient has knowledge about medical information ▪ Prefer information after receiving bad news (after progression or non-responding tumor) ▪ Perceive a lack of specific information (who to ask questions to, information for caregivers, treatment, risks and recovery) ▪ Want HCP to update them about new information	▪ Want to talk about specific topics once they are inevitable ▪ Are not sure if they would like information about the prognosis ▪ Perceive a lack of specific information (who to ask questions to, information for caregivers, treatment, risks and recovery) ▪ Have a need for information on clinical trials and treatment options	▪ Struggle with understanding what support is needed and do not want to force patients to discuss specific topics ▪ Find it difficult to determine what issues are occurring, as there can be a lot of time between consultations ▪ Struggle with supporting patients who are not open ▪ Difficult to balance between being realistic and giving hope ▪ Find it challenging not being able to give a prognosis due to the erratic disease trajectory ▪ Find it unclear what information one wants about prognosis ▪ Challenging to deal with a changing need for information throughout the disease trajectory
Patient involvement in care	▪ Would like to discuss end of life once they start to physically decline ▪ Want to give their opinion on decisions and prefer the HCP to have an advisory role ▪ Want HCP to update them about new treatments	▪ Having difficulty with being completely dependent on the HCP	▪ Having a fear of overtreating patients ▪ Having a fear of the patient only receiving treatment because the physician offers it ▪ Find it difficult to discuss and communicate with patients with cognitive problems ▪ Prefer not to advocate for getting treatment and want patients to make their own decisions
Involvement of family and friends	▪ Have a need for a conversation with the entire family when changes in the disease occur—however, this will occur more frequently among AYAs ▪ Have need for support specifically for caregivers ▪ Perceive only a focus on the patient and not on the informal caregiver	▪ Want to be involved in family conversations to know what they can arrange for the patient ▪ Do not want to draw attention to themselves, ask little questions and try not to give their opinions ▪ Perceive only a focus on the patient and not on the informal caregiver ▪ Prior to the consultation, they have to help patients to make a list of questions ▪ Would like an update once in a while ▪ Want to be involved once quality of life will be discussed	▪ Find it difficult that they are not able to provide support for informal caregivers ▪ Are struggling with patients and informal caregivers who have dysfunctional communication
Patient empowerment	▪ Want to have the final decision about their own healthcare ▪ Have a need for information regarding lifestyle to improve their own health, but perceive a lack of reimbursement and dieticians	▪ Want to focus on clinical trials and finding treatment options ▪ Have a need to be prepared for the end-of-life phase ▪ Want to be in control by knowing how to handle every situation ▪ Are seeking information online ▪ Want to know the rationale behind all treatments ▪ Are unsure if they should blindly follow the HCP	▪ Have difficulty with giving support to patients: some do not accept help, some do not take responsibility, some ask for help who do not need it ▪ Find it difficult to talk about end of life with avoidant patients
Physical support	▪ Have a need for support to get through treatment ▪ Prefer someone who regularly checks up on you ▪ Perceive a lack of support for epilepsy ▪ Experience that physical therapy is often not reimbursed or not available	▪ Have a need for someone to support the AYA to live healthier during treatment ▪ Perceive a lack of information about intimacy ▪ Would like someone to help AYA set boundaries to manage their energy-levels	-
Emotional support	▪ Have a need for a psychological track after diagnosis ▪ Want support to deal with uncertainty and their diagnosis ▪ Are scared to mention suicidal thoughts ▪ Are unsure what to discuss regarding the end of life, as the prognosis is uncertain	▪ Have a need for a psychological track after diagnosis ▪ Want support to deal with uncertainty and their diagnosis ▪ Would be helpful if appointments with a psychologist would be pre-planned, and want a separate conversation from the patient ▪ Have a need for support to process the situation, fears and worries	▪ Find it difficult not being able to resolve a patient’s situation and dealing with the same uncertainty ▪ Finding it hard to observe a patient’s physical decline ▪ Challenging that they are not able to give any certainty to the patient

## References

[1] The American Geriatrics Society Expert Panel on Person-Centered Care (2016). Person-Centered Care: A Definition and Essential Elements. J. Am. Geriatr. Soc..

[2] Scholl I., Zill J.M., Harter M., Dirmaier J. (2014). An Integrative Model of Patient-Centeredness—A Systematic Review and Concept Analysis. PLoS ONE.

[3] Grover S., Fitzpatrick A., Tabassum Azim F., Ariza-Vega P., Bellwood P., Burns J., Burton E., Fleig L., Clemson L., Hoppmann C.A. (2022). Defining and implementing patient-centered care: An umbrella view. Patient Educ. Couns..

[4] Janssen S.H.M., Vlooswijk C., Manten-Horst E., Sleeman S.H.E., Bijlsma R.M., Kaal S.E.J., Kerst J.M., Tromp J.M., Bos M.E.M.M., van der Hulle T. (2023). Learning from long-term adolescent and young adult (AYA) cancer survivors regarding their age-specific needs to improve current AYA care programs. Cancer Med..

[5] Janssen S.H.M., van der Graaf W.T.A., van der Meer D.J., Manten-Horst E., Husson O. (2021). Adolescent and Young Adult (AYA) Survivorship Practices: An Overview. Cancers.

[6] Jansen R., Kaal S.E.J., Schreuder-Cats M., Manten-Horst E. (2018). The Dutch AYA Outpatient Clinic: Support and Counselling During and After Cancer Treatment. Nursing Adolescents and Young Adults with Cancer.

[7] Graetz D., Faschiano K., Rodriguez-Galindo C., Mack J.W. (2019). Things that matter: Adolescent and young adult patients’ priorities during cancer care. Pediatr. Blood Cancer.

[8] Burgers V.W.G., van der Graaf W.T.A., van der Meer D.J., McCabe M.G., Rijneveld A.W., van den Bent M.J., Husson O. (2021). Adolescents and Young Adults Living with an Uncertain or Poor Cancer Prognosis: The “New” Lost Tribe. J. Natl. Compr. Cancer Netw..

[9] Burgers V.W.G., van den Bent M.J., Rietjens J.A.C., Roos D.C., Dickhout A., Franssen S.A., Noordoek M.J., van der Graaf W.T.A., Husson O. (2022). “Double awareness”—Adolescents and young adults coping with an uncertain or poor cancer prognosis: A qualitative study. Front. Psychol..

[10] Burgers V.W.G., van den Bent M.J., Darlington A.-S.E., van Weezel Gualtherie A.E., Compter A., Tromp J.M., Lalisang R., Kouwenhoven M., Dirven L., Harthoorn N. (2022). A qualitative study on the challenges health care professionals face when caring for adolescents and young adults with an uncertain and/or poor cancer. ESMO Open.

[11] Reuvers M.J.P., Burgers V.W.G., Vlooswijk C., Verhees B., Husson O., van der Graaf W.T.A. (2023). Same Journey, Different Paths; Caregiver Burden among Informal Caregivers of Adolescent and Young Adult Patients with an Uncertain or Poor Cancer Prognosis (UPCP). J. Clin. Med..

[12] Corbin J. (2008). Strauss A. Basics of Qualitative Research: Techniques and Procedures for Developing Grounded Theory.

[13] Braun V., Clarke V. (2019). Reflecting on reflexive thematic analysis. Qual. Res. Sport. Exerc. Health.

[14] Ritchie J., Spencer L. (2002). Qualitative data analysis for applied policy research. Analyzing Qualitative Data.

[15] QSR International Pty Ltd. (2020). NVivo. https://www.qsrinternational.com/nvivo-qualitative-data-analysis-software/home.

[16] Burgers V.W.G. (2024). The Daily Life and Care Experiences of Adolescents and Young Adults with an Uncertain or Poor Cancer Prognosis [Doctoral Dissertation, Erasmus University Rotterdam]. https://pure.eur.nl/ws/portalfiles/portal/137554378/proefschrift_vburgers_h8_under_embargo_-_65e60aaeaf6c4.pdf.

[17] Bontempo A.C. (2023). Patient attitudes toward clinicians’ communication of diagnostic uncertainty and its impact on patient trust. SSM-Qual. Res. Health.

[18] Hong H., Oh H.J. (2019). The Effects of Patient-Centered Communication: Exploring the Mediating Role of Trust in Healthcare Providers. Health Commun..

[19] Dehghani F., Barkhordari-Sharifabad M., Sedaghati-kasbakhi M., Fallahzadeh H. (2020). Effect of palliative care training on perceived self-efficacy of the nurses. BMC Palliat. Care.

[20] Mata A.N.S., de Azevedo K.P.M., Braga L.P., de Medeiros C.B.S., de Oliveira Segundo V.H., Bezerra I.N.M., Pimenta I.D.S.F., Nicolás I.M., Piuvezam G. (2021). Trainng in communication skills for self-efficacy of health professionals: A systematic review. Hum. Resour. Health.

[21] AYA Zorgnetwerk Landelijk Zorgpad AYA-Basiszorg. https://ayazorgnetwerk.nl/app/uploads/2020/07/Landelijk-zorgpad-AYA-basiszorg_versie-14-juli-2020-1.pdf.

[22] Pini S., Hackett J., Taylor S., Bekker H.L., Kite S., Bennett M.I., Ziegler L. (2021). Patient and professional experiences of palliative care referral discussions from cancer services: A qualitative interview study. Eur. J. Cancer Care.

[23] Mayland C.R., Doughty H.C., Rogers S.N., Gola A., Mason S., Hubbert C., Macareavy D., Jack B.A. (2021). A Qualitative Study Exploring Patient, Family Carer and Healthcare Professionals’ Direct Experiences and Barriers to Providing and Integrating Palliative Care for Advanced Head and Neck Cancer. J. Palliat. Care.

[24] Abdelaal M., Mosher P.J., Gupta A., Hannon B., Cameron C., Berman M., Moineddin R., Avery J., Mitchell L., Li M. (2021). Supporting the Needs of Adolescents and Young Adults: Integrated Palliative Care and Psychiatry Clinic for Adolescents and Young Adults with Cancer. Cancers.

[25] Colosimo K., Nissim R., Pos A.E., Hales S., Zimmermann C., Rodin G. (2017). “Double Awareness” in Psychotherapy for Patients Living with Advanced Cancer. J. Psychother. Integr..

[26] Rodin G., Lo C., Rydall A., Shnall J., Malfitano C., Chiu A., Panday T., Watt S., An E., Nissim R. (2018). Managing Cancer and Living Meaningfully (CALM): A Randomized Controlled Trial of a Psychological Intervention for Patients with Advanced Cancer. J. Clin. Oncol..

[27] Huiyua L., Wong C.L., Jin X., Chen J., Chong Y.Y., Bai Y. (2021). Effects of Acceptance and Commitment Therapy on health-related outcomes for patients with advanced cancer: A systematic review. Int. J. Nurs. Stud..

[28] Trevino K.M., Fasciano K., Block S., Prigerson H.G. (2012). Correlates of social support in young adults with advanced cancer. Support. Care Cancer.

[29] Smrke A., Leung B., Srikanthan A., McDonald M., Bates A., Ho C. (2020). Distinct Features of Psychosocial Distress of Adolescents and Young Adults with Cancer Compared to Adults at Diagnosis: Patient-Reported Domains of Concern. J. Adolesc. Young-Adult Oncol..

[30] Zebrack B.J., Corbett V., Embry L., Aguilar C., Meeske K.A., Hayes-Lattin B., Block R., Zeman D.T., Cole S. (2014). Psychological distress and unsatisfied need for psychosocial support in adolescent and young adult cancer patients during the first year following diagnosis. Psycho-Oncology.

[31] Gedik A., van Meerten E., Reuvers M.J.P., Husson O., van der Graaf W.T.A. (2023). The views of cancer patients of Turkish, Moroccan, Surinamese and Dutch-Caribbean descent on diagnosis, treatment and prognosis: A systematic literature review. J. Cancer Policy.

[32] Brant J.M., Silbermann M. (2021). Global Perspectives on Palliative Care for Cancer Patients: Not All Countries Are the Same. Palliat. Med..

[33] Hagerty R.G., Butow P.N., Ellis P.M., Lobb E.A., Pendlebury S.C., Leighl N., MacLeod C., Tattersall M.H.N. (2005). Communicating with realism and hope: Incurable cancer patients’ views on the disclosure of prognosis. J. Clin. Oncol..

[34] Reyes A., Galvan R., Navarro A., Velasquez M., Soriano D.R., Cabuso A.L., David J.R., Lacson M.L., Manansala N.T., Tiongco R.E. (2020). Across Generations: Defining Pedagogical Characteristics of Generation X, Y, and Z Allied Health Teachers Using Q-Methodology. Med. Sci. Educ..

